# CIBZ Regulates Mesodermal and Cardiac Differentiation of by Suppressing T and Mesp1 Expression in Mouse Embryonic Stem Cells

**DOI:** 10.1038/srep34188

**Published:** 2016-09-23

**Authors:** Tomomi Kotoku, Koji Kosaka, Miki Nishio, Yasumasa Ishida, Masashi Kawaichi, Eishou Matsuda

**Affiliations:** 1 Cell Seed Inc., Tokyo, 135-0064, Japan; 2 Division of Gene Function in Animals, Nara Institute of Science and Technology, Ikoma, 630-0192, Japan; 3 Functional Genomics and Medicine, Nara Institute of Science and Technology, Ikoma, 630-0192, Japan

## Abstract

The molecular mechanisms underlying mesodermal and cardiac specification from embryonic stem cells (ESCs) are not fully understood. Here, we showed that the BTB domain-containing zinc finger protein CIBZ is expressed in mouse ESCs but is dramatically downregulated during ESC differentiation. CIBZ deletion in ESCs induced specification toward mesoderm phenotypes and their differentiation into cardiomyocytes, whereas overexpression of CIBZ delayed these processes. During ESC differentiation, CIBZ loss-and-gain-of-function data indicate that CIBZ negatively regulates the expressions of Brachyury (T) and Mesp1, the key transcriptional factors responsible for the specification of mammalian mesoderm and cardiac progenitors, respectively. Chromatin immunoprecipitation assays showed that CIBZ binds to T and Mesp1 promoters in undifferentiated ESCs, and luciferase assays indicate that CIBZ suppresses T and Mesp1 promoters. These findings demonstrate that CIBZ is a novel regulator of mesodermal and cardiac differentiation of ESCs, and suggest that CIBZ-mediated cardiac differentiation depends on the regulation of these two genes.

Heart disease is one of the leading causes of death in the world^1^,^2^. Many heart diseases are caused by the massive loss or dysfunction of cardiomyocytes^2^. Because cardiomyocytes have a very low capacity to regenerate, many studies have focused on embryonic stem cells (ESCs) as a promising sources of cardiomyocytes to regenerate the heart. However, the molecular mechanisms that direct ESCs toward cardiac lineage remain elusive.

Mouse ESCs can differentiate into all somatic cell types in the absence of leukemia inhibitory factor (LIF)^3^,^4^. Accordingly, ESCs are widely used in studies of cardiac differentiation because this differentiation recapitulates the early cardiogenesis and gene expression profiles of cardiac development *in vivo*^5^,^6^. Transcription factors play critical roles in the regulation of ESC pluripotency and differentiation^7^,^8^ and, among these, Nanog, Oct3/4, and Sox2 are central to the maintenance of pluripotency and self-renewal^7^. In contrast, the T-box factor Brachyury (T) has been shown to regulate specification of ESCs toward a mesodermal lineage^8^. Moreover, T positively regulates the expression of Mesp1 by directly binding and activating the Mesp1 promoter^9^. Mesp1 alone has been shown to sufficiently induce cardiac specification by positively regulating downstream target genes, such as the markers of cardiac progenitors (Nkx2.5), first heart fields (Tbx5), and second heart fields (Isl1)^10^,^11^. Subsequent cardiac differentiation leads to the development of cardiac progenitor cells into mature cardiomyocytes that express structural proteins, including the cardiac troponins I and T (cTnI and cTnT) and myosin heavy chain (MHC)^12^. Although the regulatory roles of T and Mesp1 on downstream factors have previously been characterized^10^,^13^, transcriptional factor(s) that regulate T and Mesp1 transcription in ESCs remain largely unknown.

In previous studies, we identified a BTB domain-containing zinc finger protein (CIBZ; ZBTB38 in humans) that is ubiquitously expressed in mouse tissues and in various cell lines^14^,^15^. We then demonstrated that loss of CIBZ induces apoptosis in murine cells^16^, and that CIBZ negatively regulates myogenic differentiation by binding and inhibiting the myogenin promoter via its zinc finger domains^17^. Because CIBZ is highly expressed in ESCs and is critical for ESC proliferation^18^, we hypothesized that CIBZ plays a role in the regulation of ESC differentiation.

To address whether the expression of CIBZ influences ESC differentiation, we used CIBZ knockout ESCs and CIBZ overexpressed ESCs, respectively, and observed their differentiation potential *in vitro*. ESCs in suspension culture can form three-dimensional aggregates known as embryonic bodies (EBs), which can differentiate into descendants of the three germ layers, even though very few of these cells are cardiomyocytes^6^. The conventional hanging-drop approach has been widely used to form EBs^19^,^20^ but has disadvantages for mass preparation because of limited sphere sizes. Recently, rotary suspension cultures have been used to generate EBs with larger and more homogeneous spheres^21^,^22^,^23^. Using this method, CIBZ loss-and-gain-of-function data has shown that CIBZ suppresses differentiation of ESCs toward mesodermal and cardiac lineages. Moreover, present data indicate that CIBZ negatively regulates the expression of T and Mesp1 during ESC differentiation. Hence, the regulation of ESCs toward mesodermal and cardiac differentiation by CIBZ is dependent, at least in part, on the regulation of T and Mesp1 genes.

## Results

### CIBZ Protein is Downregulated during ESC Differentiation and Loss of CIBZ Induces Mesodermal Genes in ESCs

**Figure 1:**
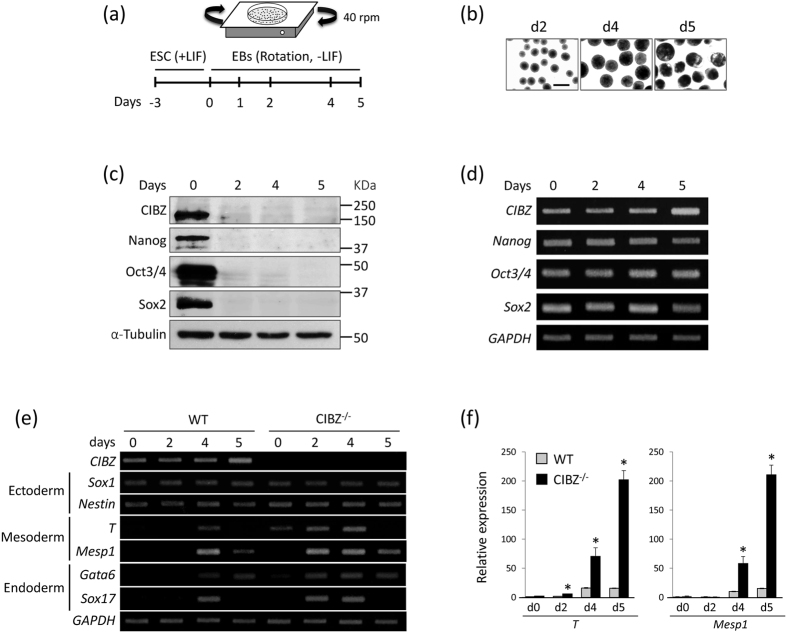
The CIBZ protein is downregulated during the formation of embryonic bodies (EB) and the loss of CIBZ triggers mesodermal gene expression: **(a)** Schematic of the rotary suspension culture used to differentiate ESCs; **(b)**, Bright-field microscopy of wild type (WT) EBs formed in rotary orbital suspension cultures on days 2, 4, and 5; Scale bar = 500 μm; **(c**,**d)** Expression of the indicated proteins and genes in WT embryonic stem cells (ESCs) were examined using Western blotting (**c**) and semi-quantitative PCR (**d**) with α-tubulin and GAPDH as loading controls, respectively; **(e)** Expression levels of indicated genes in WT and CIBZ^−/−^ ESCs were detected using semi-quantitative PCR with GAPDH as a loading control; **(f)** Real-time PCR (qPCR) showing mRNA levels of T and Mesp1 in WT and CIBZ^−/−^ ESCs on days 0–5 of differentiation. Data are presented in columns and were normalized to GAPDH expression; *p < 0.05.

To investigate the roles of CIBZ, we monitored its expression during ESC differentiation. Rotary suspension cultures lacking LIF and feeder cells were used to induce formation of uniform populations of EBs from ESCs (Fig. 1a,b). As shown in Fig. 1c, expression levels of the pluripotent marker proteins Nanog, SOX2, and Oct3/4, and CIBZ were dramatically decreased during EB formation from days 2–5. However, corresponding mRNA expression levels remained relatively stable during EB formation (Fig. 1d), indicating the predominance of posttranscriptional regulation of these proteins, as described previously^24^,^25^.

To determine whether loss of CIBZ affects ESC differentiation, we used CIBZ knockout (CIBZ^−/−^) ESC line, which was maintained in the undifferentiated state, as described previously^18^, and the effects of CIBZ knockout were determined using semi-quantitative PCR. These experiments showed no changes in Nanog, Sox2, and Oct3/4 expression in the absence of CIBZ during ESC differentiation (Supplementary Fig. S1), suggesting no regulatory effects of CIBZ on these pluripotent genes. In contrast, loss of CIBZ in ESCs induced mesodermal T, cardiac mesodermal Mesp1, and the endodermal genes Gata6 and Sox17, but did not greatly affect expression levels of the ectoderm markers Sox1 and Nestin during EB formation from days 2–5 (Fig. 1e). In addition, real-time PCR (qPCR) data confirmed the induction of T and Mesp1 in CIBZ^−/−^ EBs compared with WT EBs (Fig. 1f). Moreover, siRNA-mediated transient knockdown of CIBZ in ESCs led to the induction of T and Mesp1 genes during ESC differentiation on days 3–5 (Supplementary Fig. S2). These data indicate that loss of CIBZ in ESCs induces T and Mesp1 expression during differentiation. Because Mesp1 is a known master regulator of cardiac differentiation, we hypothesized that CIBZ loss promotes ESC differentiation toward the cardiac lineage.

### Loss of CIBZ Accelerates ESC Differentiation toward Cardiac Maturation

**Figure 2:**
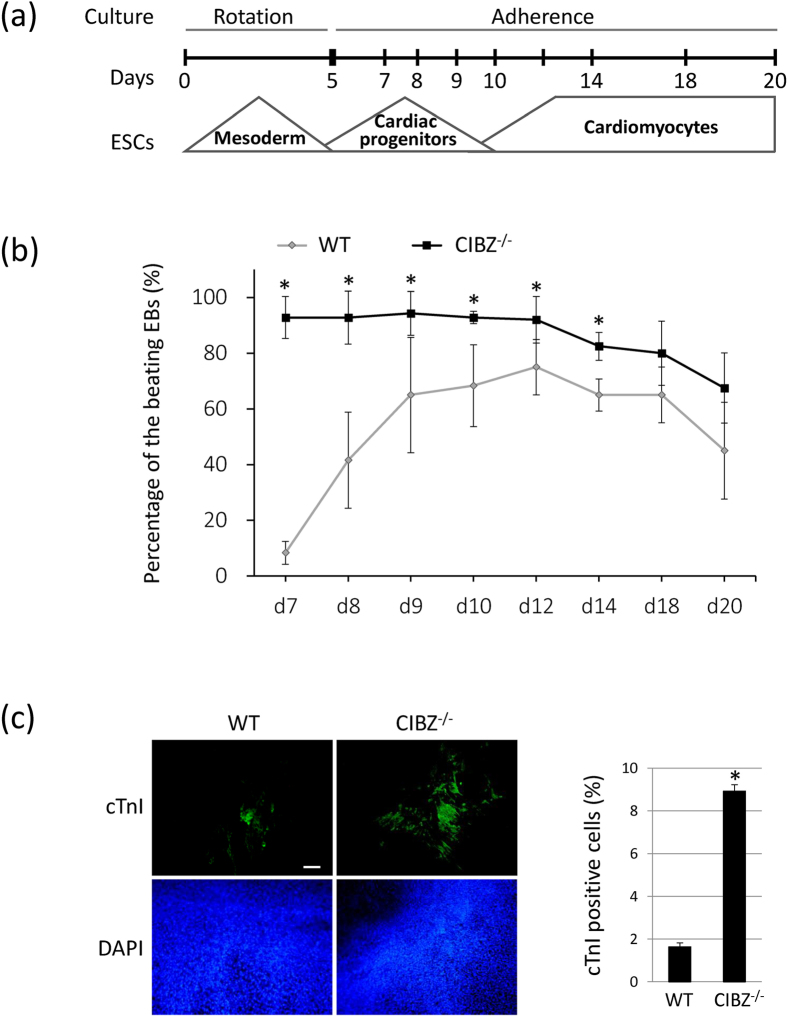
Loss of CIBZ induces ESC differentiation towards cardiac maturation: **(a)** Schematic presentation of *in vitro* strategies for cardiomyocyte differentiation from ESCs; **(b)** The incidence of spontaneously beating EBs from ESCs was quantified at the indicated time points during differentiation; N = 25, *p < 0.05; **(c)** Immunofluorescent analysis of cardiac Troponin I (green) on day 10 EBs; Scale bar = 20 μm; Left panel, cTnI positive cells and DAPI-stained nuclei; Right panel, quantitative evaluations of cTnI positive cardiomyocytes in EBs. Presented data are representative of 15 randomly chosen fields; *p < 0.05.

To confirm cardiogenesis in CIBZ deficient ESCs, spontaneously beating ESC-derived cardiomyocytes were observed under a microscope. To increase cardiac differentiation efficiency^26^, a single 5-day-old EB in suspension culture was transferred to a gelatin-coated 24-well plate for further adherent culture (Fig. 2a). Subsequently, approximately 10% of WT EBs started to beat spontaneously at day 7 (Fig. 2b), and the incidence of beating EBs increased gradually to 60–80% by day 14. In contrast, approximately 90% of CIBZ^−/−^ EBs started to beat at day 7 (Fig. 2b), and more beating areas were observed on day 10 (Supplementary Video 1–2). Moreover, in immunofluorescence confocal microscopy analyses, areas of CIBZ^−/−^ EBs expressing the cardiomyocyte marker cTnI were approximately 5.6-fold larger than those of WT EBs at day 10 (Fig. 2c). These observations demonstrate that loss of CIBZ promotes cardiomyocyte maturation of ESCs.

### Loss of CIBZ Induces the Expression of Genes that Drive ESC Differentiation toward the Cardiac Lineage and Cardiac Maturation

**Figure 3:**
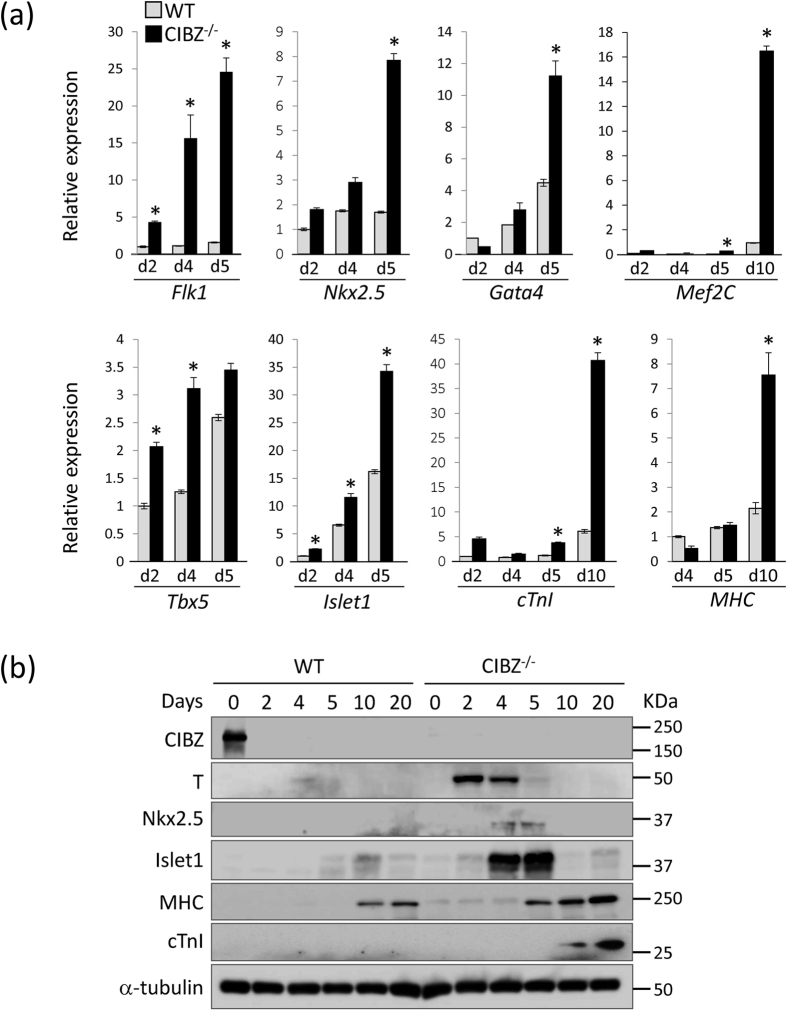
Loss of CIBZ induces the expression of cardiac genes during ESC differentiation: **(a)** mRNA levels of the indicated genes in WT and CIBZ^−/−^ ESCs were determined using qPCR. Data are presented in columns and were normalized to GAPDH expression; *p < 0.05; **(b)** Protein expression of indicated proteins in WT and CIBZ^−/−^ ESCs was determined using Western blotting with α-tubulin as a loading control.

In further experiments, we determined whether loss of CIBZ induces the expression of genes that have been associated with cardiac differentiation. The ensuing qPCR data showed that loss of CIBZ in ESCs induced mRNA expression of the markers of cardiac progenitors (Flk1, Nkx2.5, Gata4, and Mef2C), the first heart field (Tbx5), the second heart field (Islet1), and the cardiac myocyte (cTnI and MHC) during ESC differentiation from days 2 to 10 (Fig. 3a). Subsequent immunoblot analysis confirmed the protein induction of T, Nkx2.5, Islet1, MHC, and cTnI proteins in CIBZ^−/−^ EBs in comparison with those in WT EBs during ESC differentiation (Fig. 3b). These findings indicate that loss of CIBZ induces robust expression of both early and late stage cardiac cell markers, promoting ESC differentiation into cardiomyocytes.

### Overexpressed CIBZ Inhibits ESC Differentiation Toward Mesoderm and Cardiac Lineages

**Figure 4:**
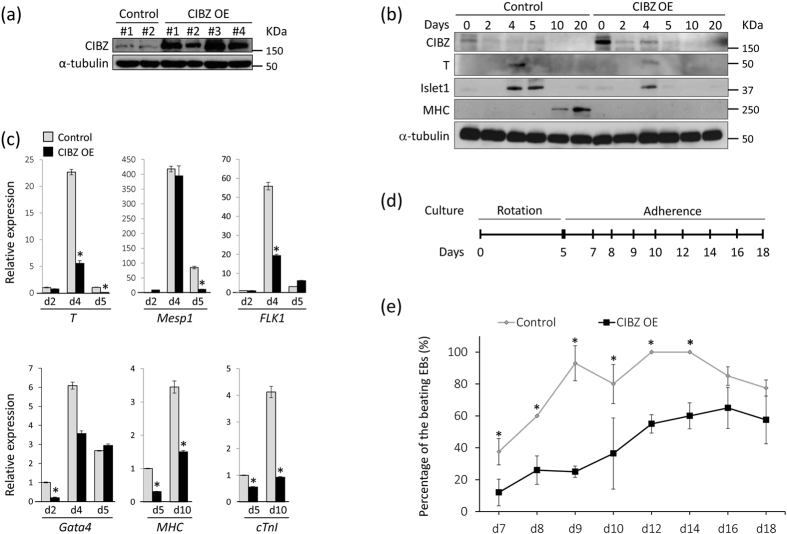
Constitutive ectopic expression of CIBZ suppresses ESC differentiation: **(a)** Western blotting analyses of stable pEF1α (control) and pEF1α-CIBZ (CIBZ OE) transfected ESCs. Control #1 and CIBZ OE #3 clones were chosen for subsequent studies; OE, overexpression; **(b,c)** Expression levels of the indicated proteins and mRNAs in WT and CIBZ OE EBs were determined using immunoblotting (**b**) and qPCR (**c**) respectively; Data from qPCR experiments (**c**) were normalized to the expression of GAPDH; *p < 0.05; **(d)** A schematic presentation of differentiation strategies for induction of cardiomyocyte differentiation in ESCs; **(e)** The incidence of spontaneously beating EBs was quantified at the indicated time points during differentiation; N = 20, *p < 0.05.

To investigate the reciprocal effects of CIBZ overexpression on ESC differentiation, we generated ESC lines that stably overexpress CIBZ (CIBZ-OE) under the control of the EF1α promoter, which is a stable and strong promoter in ESCs^27^. CIBZ-OE clone #3 was used for the subsequent studies because it showed the highest expression of CIBZ protein among the four clones isolated (Fig. 4a). Immunoblotting analyses of CIBZ overexpressing ESCs corroborated decreases in T, Islet1, MHC, and cTnI protein expression compared to the control ESCs during differentiation (Fig. 4b). qPCR and semi-quantitative PCR data showed that CIBZ overexpression in ESCs inhibits the expression of the mesoderm and cardiac progenitor markers T, Mesp1, Flk1, and Gata4 from days 2 to 5, and inhibits the cardiac myocyte markers MHC and cTnI from days 5 to 10 in comparison with those in control cells (Fig. 4c; Supplementary Fig. S3). Taken together, these data suggest that overexpression of CIBZ inhibits ESC differentiation toward mature cardiomyocytes. To confirm this, a single 5-day-old EB in suspension culture was transferred to a gelatin-coated 24-well plate for further adherent culture to enhance cardiac differentiation (Fig. 4d). As shown in Fig. 4e, numbers of beating EBs were significantly suppressed in CIBZ overexpressing ESCs on days 7–14, confirming that CIBZ overexpression inhibits ESC differentiation toward cardiac maturation, probably by inhibiting T and Mesp1 transcription.

### CIBZ Binds to and Represses T and Mesp1 Promoters

**Figure 5:**
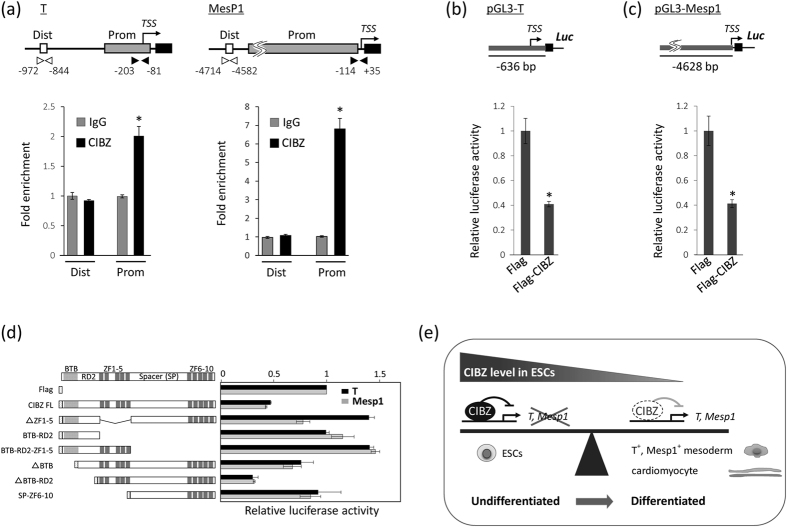
CIBZ binds and represses T and Mesp1 promoters: **(a)** A schematic of the proximal regions of the T and Mesp1 promoters (upper panel); ChIP assays were performed using an anti-CIBZ antibody in undifferentiated ESCs. Non-specific IgG was used as a negative control. Input DNA (2.5%), IgG-precipitated DNA, and CIBZ-immunoprecipitated DNA were amplified using primers for indicated promoter (Prom) and distal regions (Dist) as indicated; TSS, transcriptional start site. qPCR data are shown as fold enrichment relative to input control, which was set to 1. N = 3, *p < 0.05; **(b**,**c)** Schematic representation of pGL3-T and pGL3-Mesp1 showing promoter lengths; pGL3 vector, pGL3-T, or pGL3-Mesp1 were cotransfected with expression vectors for Flag or Flag-CIBZ into HEK293T cells, and pRL-TK was contransfected as an internal control. Luciferase activities were determined relative to that of the pGL3 vector; N = 6, *P < 0.05; TSS, transcriptional start site; **(d)** CIBZ ZF1-5 is required for repression of T and Mesp1 promoters; Schematic representation of CIBZ deletion mutants fused to Flag (left), and their repressor activities on the pGL3-T or pGL3-Mesp1 reporters (right); **(e)** Proposed functions of CIBZ during ESC differentiation. Loss of CIBZ in ESCs is a prerequisite for specification toward mesoderm cells and differentiation into cardiomyocytes.

CIBZ loss-and-gain-of-function data suggested that CIBZ regulates T and Mesp1 by directly binding to their promoters in ESCs. To confirm this, ChIP assays were performed after precipitating chromatin from undifferentiated ESCs using an anti-CIBZ antibody or a negative control (normal rabbit IgG), as described previously^17^. These experiments (Fig. 5a) showed that CIBZ binds to T and Mesp1 promoter regions, but does not bind to the evolutionarily conserved regions upstream of these two promoters. In addition, CIBZ failed to bind to Flk1, Nkx2.5, and Gata4 promoters (Supplementary Fig. S4). These results indicate that CIBZ binds specifically to the T and Mesp1 promoters in ESCs.

To confirm that CIBZ directly suppresses T and Mesp1 transcription, a 636-bp T promoter region^28^,^29^ and a 4.6-Kb Mesp1 promoter region^30^ were cloned into the pGL3 luciferase reporter vector to generate pGL3-T and pGL3-Mesp1 reporters (see Methods). Subsequent luciferase assays showed that Flag-CIBZ inhibited the expression of pGL3-T and pGL3-Mesp1 reporters by about 2.5-fold when compared with Flag alone in HEK293T cells, which express CIBZ at very low levels (Fig. 5b,c). To identify domain(s) of CIBZ that associate with these repressive activities, a series of Flag-CIBZ deletion mutants were generated as described previously^14^. In subsequent luciferase assays (Fig. 5d), mutants lacking ZF1-5 (ΔZF1-5, BTB-RD2, and SP-ZF6-10) failed to inhibit pGL3-T and pGL3-Mesp1 constructs, indicating that this domain is required for the repressive activities of CIBZ. In addition, ZF1-5 and the C-terminal mutant ΔBTB-RD2 displayed full repressive activities, indicating these domains are sufficient for T and Mesp1 promoter repression by CIBZ.

Taken together, these findings show that CIBZ binds to, and represses, T and Mesp1 promoters, and strongly suggest that CIBZ-mediated mesodermal and cardiac differentiation of ESCs depends on the regulation of these two genes.

## Discussion

The data presented here demonstrate that decreased expression of CIBZ is a prerequisite for ESC specification toward mesoderm cells and differentiation into cardiomyocytes (Fig. 5e).

Recent studies implicate protein expression of Oct3/4 but not Nanog in mesodermal commitment of ESCs^31^,^32^,^33^. For example, twofold forced expression of Oct3/4 in ESCs resulted in induction of T and Mesp1, which have been associated with ESC commitment to mesodermal and cardiac lineage. In agreement with this, our data showed that CIBZ deletion in the present ESCs led to increases in Oct3/4 protein expression compared with that in WT ESCs on day 2 of differentiation (Supplementary Fig. S5a). Thus, we propose a model (Fig. 5e) in which transcription of T and Mesp1 is silenced by CIBZ in undifferentiated ESCs, and T expression is induced by decreases in CIBZ protein levels, likely as a result of increased signals (canonical Wnt and Activin, etc)^34^,^35^,^36^ upon differentiation. Upon further differentiation, loss of CIBZ enables T and Oct3/4 to activate Mesp1, which then commits cells to cardiac differentiation. Taken together with observations that overexpression of CIBZ inhibits the expression of T and Mesp1 during ESC differentiation and suppresses ESC specification toward mesodermal and cardiac lineages, the present data suggest that CIBZ functions as a gatekeeper of ESC pluripotency.

Our data showed that loss of CIBZ in undifferentiated ESCs failed to increase protein expression of Oct3/4, rather leading to slight decreases, and CIBZ overexpression did not affect the expression of Oct3/4 (Supplementary Fig. S5). While forced expression of Oct3/4 enhances ESC differentiation into mesoderm and endoderm lineages^31^,^32^, decreased Oct3/4 levels lead to ESC differentiation toward trophectoderm lineages^31^. Molecularly, Oct3/4 reportedly activates Mesp1 by binding to the Mesp1 promoter but not to the T promoter, and the former cascade required canonical Wnt signaling^37^. By contrast, our data showed that Wnt (Wnt3a or LiCl) or Activin is not necessarily required for CIBZ-mediated inhibition of T and Mesp1, although these signals did activate pGL3-T and pGL3-Mesp1 expression in HEK29l cells (data not shown). Further studies are needed to explore whether TGFβ or/and bone morphogenetic proteins (BMPs), which also regulate cardiac differentiation in ESCs^32^,^36^,^38^,^39^,^40^, affect CIBZ-mediated repression of T and Mesp1 promoters; as well as regulate CIBZ protein levels in ESCs.

The data presented here indicate that CIBZ suppresses T and Mesp1 promoters (Fig. 5b–d), probably due to either direct promoter binding or protein–protein interactions. Because CIBZ is a methyl-CpG binding protein^17^, it may act by DNA methylation. Our previous ChIP data showed that treatment of myoblast cells with the demethylation reagent 5-aza-dC abolished CIBZ binding to the myogenin promoter, and CIBZ suppressed the myogenin promoter by its promoter methylation *in vitro*^17^. Our data here showed that 5-aza-dC treatments increased mRNA expression of T in ESCs (Supplementary Fig. S6a), thereby suggesting that DNA methylation is also involved in the regulation of T expression in ESCs, in agreement with correlations between T promoter demethylation with its upregulation in stem and somatic cells^41^,^42^. However, 5-aza-dC treatments did not decrease binding of CIBZ to T and Mesp1 promoters, thereby suggesting that DNA methylation was not required for binding of CIBZ to T and Mesp1 promoters or for the ensuing inhibition of T and Mesp1 promoters *in vitro* (Supplementary Fig. 6b,c). Moreover, ZBTB38 preferentially binds to methylated CGCCAT or GCGGTA motifs^41^,^42^, which are present in the myogenin promoter region, but not in T or Mesp1 promoter regions, suggesting that CIBZ binds to the undescribed sites of the T and Mesp1 promoters. Future studies are required to identify the consensus binding sequence of CIBZ using systematic evolution of ligands by exponential enrichment (SELEX)^43^, or protein binding microarrays (PBMs)^44^,^45^, and to identify the binding partners of CIBZ in ESCs using mass spectrometry.

In initial experiments, the CIBZ protein was downregulated without changes in mRNA expression during EB formation (Fig. 1c,d), indicating the predominance of post-transcriptional regulatory mechanisms. The ubiquitin–proteasome pathway is considered central to protein turnover during stem cell differentiation^46^. However, pretreatment of ESCs with the proteasome inhibitor MG132 reportedly inhibited Nanog protein degradation during differentiation on day 2^24^,^47^, but failed to rescue CIBZ protein expression (Supplementary Fig. S7), although the treating time and concentration of MG132 are required for optimization. Thus, the ubiquitin–proteasome pathway may also not be necessary for the downregulation of CIBZ protein expression. Taken together, these observations warrant future mechanistic studies using miRNAs that modulate gene expression by inhibiting protein translation^48^,^49^, and assessments of the ensuing regulation of CIBZ protein during ESC differentiation.

ESCs have been considered as a reliable source of cells for the treatment of various heart diseases. However, differentiating ESC cultures contain very low percentages of beating cardiomyocytes. The present experiments show that CIBZ suppresses cardiac differentiation of ESCs by inhibiting T and Mesp1 and, thus, extend our understanding of the molecular pathways that guide ESC differentiation toward mesodermal and cardiomyocyte lineages. Future studies are warranted to identify clinically active CIBZ antagonists that promote cardiomyocyte differentiation for the treatment of heart disease.

## Methods

### Statement

All experiments were approved by Nara Institute of Science and Technology and were carried out in accordance with guidelines that were established by the Science Council of Japan.

### Cell Culture and EB Formation

Mouse RF8 ESCs^50^ derived from 129/TerSv mice were cultured as described previously^15^. Briefly, ESCs were maintained on mitomycin C-treated SNL-STO cells^51^ hich express LIF in standard ESC culture medium [DMEM (Nacalai Tesque), 15% ESC-qualified fetal bovine serum (FBS, Sigma-Aldrich), 2-mM L-glutamine, 100-μM nonessential amino acids, 1% penicillin and streptomycin, and 0.1-μM β-mercaptoethanol]. Rotary suspension cultures were used to generate EBs according to previously described methods^23^. Briefly, undifferentiated ESCs were trypsinized and cultured in ES medium at 2 × 10^6^ cells per 10 ml in non-adherent Petri dishes on an orbital shaker (NS-LR, AS ONE) at 37 °C in 5% CO_2_. To ensure consistency, the speed was set at 40 rpm for the entire suspension culture period. Culture media was changed every other day, and EBs were collected days 2–6 after differentiation. To promote cardiac differentiation^26^, EBs from day-5 suspension cultures were collected and individually reseeded onto 0.1% gelatin-coated 24-well tissue culture dishes for visual inspection, video recording, and immunofluorescence or were plated onto 0.1% gelatin-coated 10-cm dishes at about 100 EBs/dish for RNA and protein extraction. The development of beating foci within EB colonies in 24-well dishes was observed everyday using a phase-contrast microscope and beating was recorded using a video camera. Percentages of beating EBs relative to total numbers of plated EBs were calculated, and at least 20 EBs per experimental sample were counted. All the experiments were performed 3–6 times.

### Semi-quantitative and real-time PCR (qPCR)

Semi-quantitative PCR was performed as described previously^14^. Primer sequences were confirmed as unique using the non-redundant NCBI database and are listed in the Table S1. Primer annealing was performed at 58–60 °C for all primer sets. Reaction products were then separated on 2% agarose gels and visualized using ethidium bromide staining. To identify PCR products, single bands of expected sizes were excised from gels and sequenced. GAPDH mRNA expression was used as an internal control.

Real-time PCR (qPCR) was performed using a LightCycler^®^ 96 System (Roche Diagnostics) with the Thunderbird SYBR Green PCR Mix (Toyobo), following the manufacturer’s instructions. Subsequently, 2-μl aliquots of all cDNA samples were analyzed in triplicate on 96-well optical PCR plates (Roche Diagnostics). The qPCR protocol included preincubation at 95 °C for one minute to activate Taq DNA polymerase and 45 amplification cycles of 95 °C for 15 s, 59 °C for 30 s, and 72 °C for 30 s, followed by a melting step from 65 °C to 97 °C over 60 s under continuous fluorescence measurement, and final cooling to 37 °C. Amplicons with expected molecular weights were identified using gel electrophoresis with cDNA-free samples as negative controls. GAPDH was used as the reference gene and all analyses were performed using the ΔΔCt method with Roche LightCycler 96 system software. Primer sequences are listed in the Table S2.

### Western Blotting

Western blotting was performed as described previously^52^. Briefly, protein lysates were prepared in RIPA buffer supplemented with complete protease inhibitor (Roche Applied Science). Proteins were then separated on 6–15% SDS-PAGE, transferred onto PVDF membranes, and probed with anti-CIBZ [amino acids 1184–1197 (EQKDDIKAFAENVL) of CIBZ]^17^, anti-Oct3/4 (MAB1759, R&D Systems), anti-Sox2 (S1451, Sigma-Aldrich), anti-Nanog (AB5731, Millipore), anti-α-tubulin (clone DM 1A, Sigma-Aldrich), anti-Brachyury (sc-17743, Santa Cruz Biotechnology), anti-Nkx-2.5 (sc-8697, Santa Cruz Biotechnology), anti-Islet 1 (ab109517, Abcam), anti-cTnI (ab19615, Abcam), and anti-MHC (clone MF20, Developmental Studies Hybridoma Bank) antibodies. HRP-conjugated anti-mouse or anti-rabbit IgG (Cell Signaling) were used as secondary antibodies.

### Plasmid Preparation and Generation of CIBZ Stable Cell Lines

To clone the plasmid pEF1α-IRES-CIBZ, full-length CIBZ fragments were digested with BamHI and ApaI from pcDNA3-CIBZ^14^,^16^ and were then ligated into the corresponding sites of pEF1α-IRES (Clontech). DNA sequences were then verified using BigDye and an automated sequencer (ABI PRISM3100). ESCs (5 × 10^5^ cells in 10-cm plates) were then transfected with 10 μg of the desired constructs using TransFast reagent (Promega) according to the manufacturer’s protocol. Neomycin-resistant clones were picked after 12–14 days of G418 (200 μg/ml) selection and were propagated in the same ESC medium.

### Immunofluorescence Microscopy

Immunofluorescence analyses were performed as described previously^53^. Briefly, EBs were fixed in 3.7% paraformaldehyde/PBS for 30 min at room temperature and were then permeabilized with 0.2% Triton-X100/PBS for 30 min. Cells were then incubated with anti-cTnI antibody (ab19615, Abcam) followed by CF488-conjugated donkey anti-mouse IgG (Biotium). Subsequently, DNA was counterstained with 4′,6-diamidino-2-phenylindole (DAPI, Sigma), and confocal microscopy analyses were performed using a Zeiss LSM510 microscope. Images were captured using Zeiss LSM510 v3.0 software and were processed using Adobe Photoshop 5.5. All staining experiments were repeated at least three times.

### Chromatin Immunoprecipitation (ChIP) Assays and ChIP-qPCR

ChIP assays were performed as described previously^17^. Briefly, ESCs were grown to subconfluence on 10-cm plates, and after crosslinking for 30 min with 1% formaldehyde, glycine was added to a final concentration of 0.125 M, and the cells were washed three times in ice-cold PBS and harvested using SDS-lysis buffer. Chromatin lysates were then sonicated on ice to an average DNA length of 500 bp. After preclearing the lysates with Protein G Sepharose beads, anti-CIBZ antibody^17^ was used to immunoprecipitate protein-DNA complexes and Preimmune IgG was used as a negative control. Precipitated DNA was subjected to qPCR using primers (Table S3) for promoter regions (prom) and distal regions.

### Reporter Constructs and Luciferase Assays

Genomic DNA from RF8 ESCs was amplified using PCR with primers for T and Mesp1 (Table S4) that incorporated the restriction sites MluI and HindIII. Fragments of T^28^ (492 to +144 bp) and Mesp1^30^ (−4557 to +71) were generated and ligated into pre-digested pGL3 vectors to produce pGL3-T and pGL-3-Mesp1 constructs. DNA sequences were then verified using an automated sequencer (ABI PRISM3130). Subsequently, HEK293T cells were cultured in DMEM (Nacalai Tesque) supplemented with 10% fetal bovine serum (Sigma-Aldrich) and luciferase assays were performed as described previously^52^. Briefly, cells were transfected with 0–250 ng of expression plasmids for Flag or Flag-fusion proteins and 100 ng of firefly luciferase reporters, pGL3 vector, pGL3-T, or pGL3-Mesp1. Four nanograms of pRL-TK reporter (Promega) was used as an internal control and total amounts of expression plasmids were standardized to that of the empty plasmid pcDNA3. Firefly luciferase activity was normalized to transfection efficiency according to the luciferase activity of the internal control. All experiments were performed in duplicate and data are presented as means ± standard deviations (SD) of three-six independent experiments.

### Statistical analysis

Unless stated otherwise, data are presented as means ± SD of three independent experiments. Statistical analyses were performed with a nonparametric Mann–Whitney *U* test for the incidence of beating EBs, and two-unpaired Student’s *t*-test for the other experiments. Differences were considered significant when *p < 0.05.

## Additional Information

**How to cite this article**: Kotoku, T. *et al*. CIBZ Regulates Mesodermal and Cardiac Differentiation of by Suppressing T and Mesp1 Expression in Mouse Embryonic Stem Cells. *Sci. Rep.* **6**, 34188; doi: 10.1038/srep34188 (2016).

## Supplementary Material

Supplementary Information

Supplementary Video

Supplementary Video
