# Supplementary material for: CIBZ Regulates Mesodermal and Cardiac Differentiation of by Suppressing T and Mesp1 Expression in Mouse Embryonic Stem Cells
Source: Sci Rep. 2016 Sep 23;6:34188. doi: 10.1038/srep34188 (PMC5034229; doi:10.1038/srep34188)
Supplement: Supplementary Information [file srep34188-s1.pdf]

CIBZ Regulates Mesodermal and Cardiac Differentiation of by Suppressing T and Mesp1 Expression in Mouse Embryonic Stem Cells

Tomomi Kotoku[1], Koji Kosaka[2], Miki Nishio[3], Yasumasa Ishida[3], Masashi Kawaichi[2] and Eishou Matsuda[2,*]

Supplementary Supporting Information

Supplementary Methods
Supplementary Figure Legends
Supplementary Figures
Supplementary tables
Supplementary video legends

Supplementary Methods

siRNA and Transient Transfection

Mouse ESCs were transfected with 50-nM CIBZ-specific or scrambled negative control dicer substrate siRNA duplexes (Integrated DNA Technologies) as described previously[18]. Transfection with siRNA was performed using INTERFERin reagent (Polyplus Transfection) according to the manufacturer's instructions. Subsequently, siRNA-transfected cells were cultured for 3 days and were then cultured under rotary suspension conditions for an additional 2 days.

Chromatin Immunoprecipitation (ChIP) Assays

ChIP assays were performed as described previously[17]. Amplification of immunoprecipitated DNA was achieved using Blend Tag plus DNA polymerase (Toyobo), after annealing primers (Table S3) for promoter regions (prom) and distal regions (dist) at 55°C. All experiments were repeated three times.

Supplementary Figure Legends

S1. Expression of Nanog, Oct3/4, and Sox2 in WT and CIBZ$^{-/-}$ ESCs during ESC differentiation; mRNA levels of the indicated genes were determined using semi-quantitative PCR with GAPDH as a loading control.

S2. Knockdown of CIBZ in ESCs upregulates T and Mesp1 during ESC differentiation; siRNA duplexes were used to reduce CIBZ expression in ESCs. Three days after transfection with 50-nM CIBZ siRNA or 50-nM scrambled negative siRNA (control), ESCs were cultured under rotary suspension conditions for an additional 2 days (day 5), and expression of the indicated genes was determined using semi-quantitative qPCR with GAPDH as a loading control.

S3. CIBZ overexpression in ESCs suppresses cardiac genes during ESC differentiation; mRNA levels of the indicated genes were determined using semi-quantitative qPCR with GAPDH as a loading control.

S4. CIBZ binds to the Mesp1 promoter region but fails to bind to promoter regions of Flk1, Nkx2.5, and Gata4 genes in ESCs; ChIP assays were performed using an anti-CIBZ antibody in undifferentiated ESCs. Non-specific IgG was used as a negative control. Input DNA (1%), IgG-precipitated DNA, and CIBZ-immunoprecipitated DNA were amplified using primers for the indicated promoter regions.

S5. Western blotting analysis of Nanog, Oct3/4, and Sox2 expression during ESC differentiation; Expression levels of the indicated proteins in WT and CIBZ$^{-/-}$ ESCs (a), and in pEF1α (control) and pEF1α-CIBZ (CIBZ OE) transfected ESCs (b); α-tubulin was used as a loading control.

S6. Treatment of ESCs with 5-aza-dC failed to abolish binding of CIBZ to T and Mesp1 promoter regions:
(a), Effects of 5-aza-dC treatment on ESCs; ESCs were treated with 5-aza-dC (0.25 μM or 0.5 μM) for 24 hours and expression of indicated genes and proteins was determined using semi-quantitative PCR (left panel) and Western blotting (right panel) with β-actin and α-tubulin as loading controls, respectively; **(b),** Schematic representation of 5′ regions of T and Mesp1 promoters (BLAT search genome, UCSC Genome Bioinformatics); Dist, distal; Prom, promoter; **(c),** ChIP assays were performed using an anti-CIBZ antibody in untreated (upper panel) and 5-aza-dC (0.25 μM) treated ESCs (lower panel). Input DNA (2.5%), IgG-precipitated DNA, and CIBZ-immunoprecipitated DNA were amplified using indicated primers for T (left panel) and Mesp1 (right panel).

S7. MG132 treatments of ESCs failed to rescue CIBZ protein expression. ESCs (denoted as EB d0) were trypsinized, were treated with MG132 (2 and 5 μM) or DMSO or vehicle (“-”), and were then cultured in ES medium on non-adherent Petri dishes on an orbital shaker for 2 days. Expression of indicated proteins was determined using Western blotting with α-tubulin as a loading control.

Supplementary Fig. S1

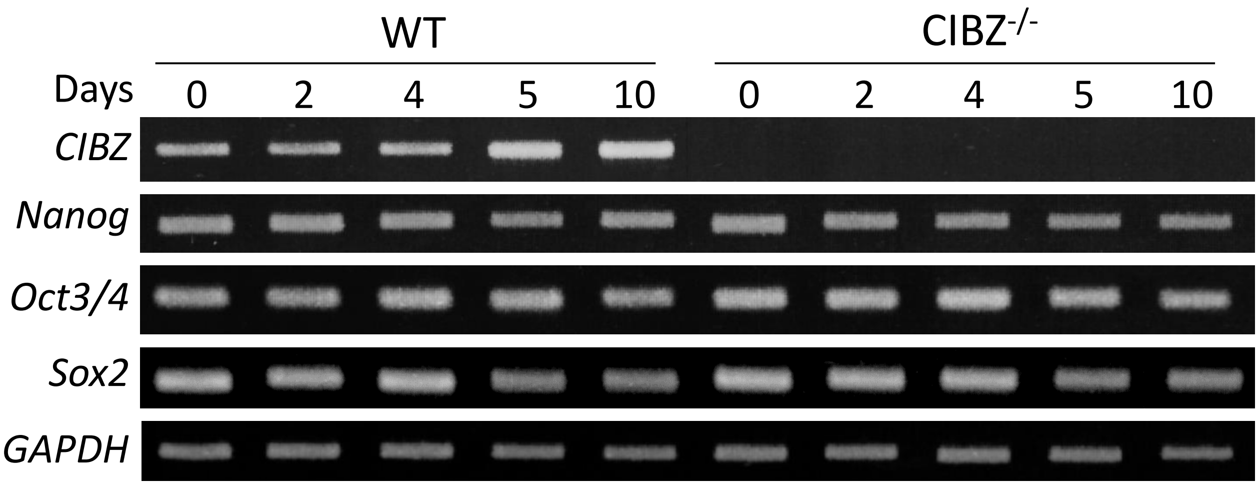

Supplementary Fig. S2

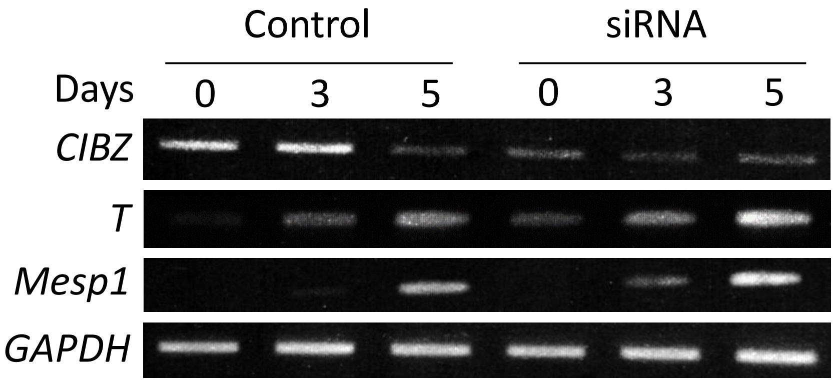

Supplementary Fig. S3

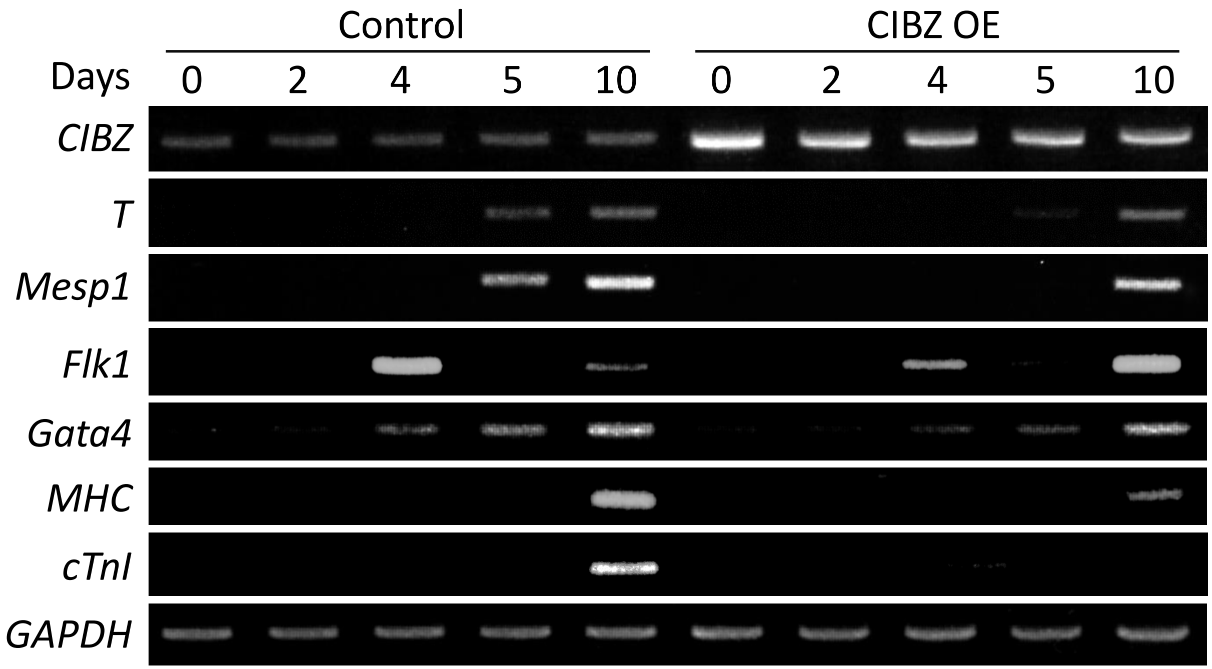

Supplementary Fig. S4

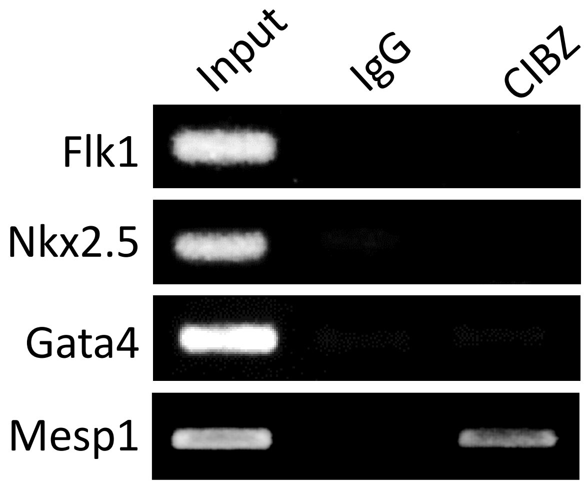

Supplementary Fig. S5

a

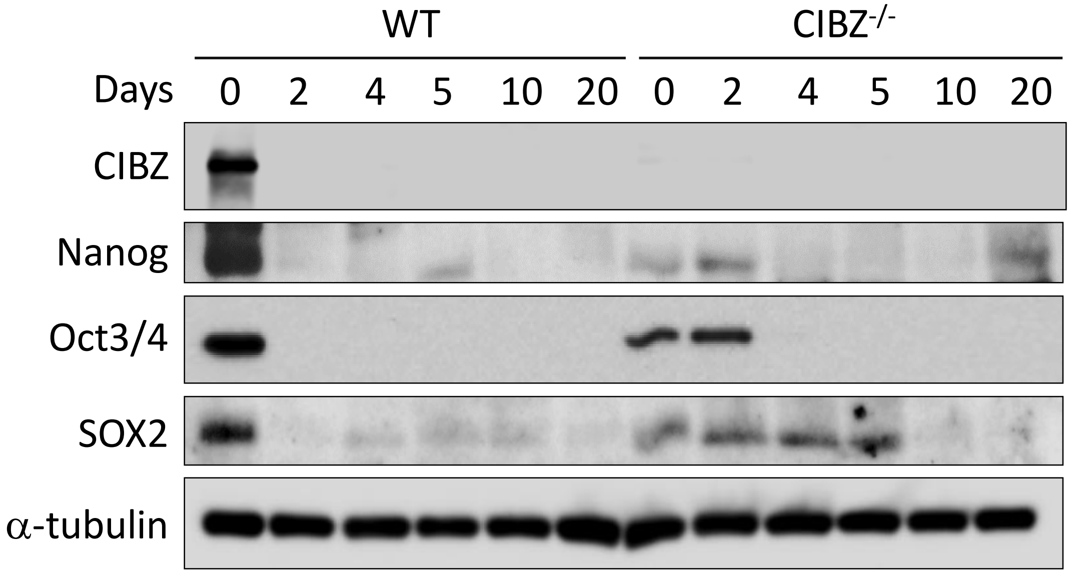

b

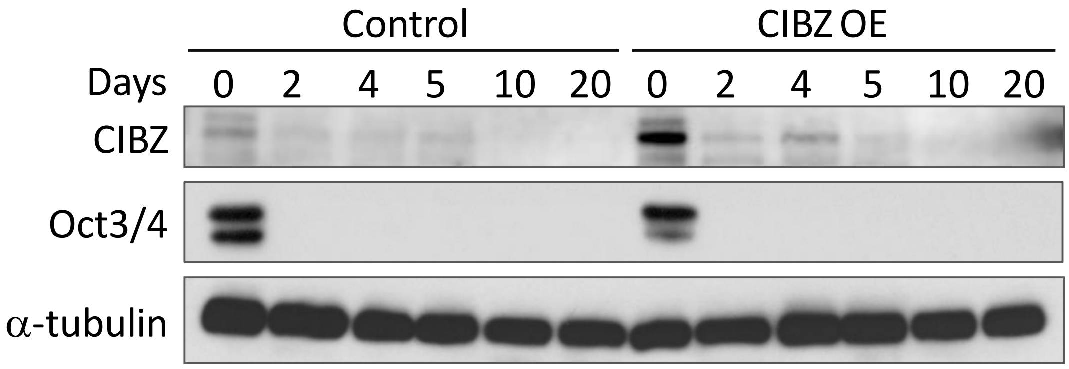

Supplementary Fig. S6

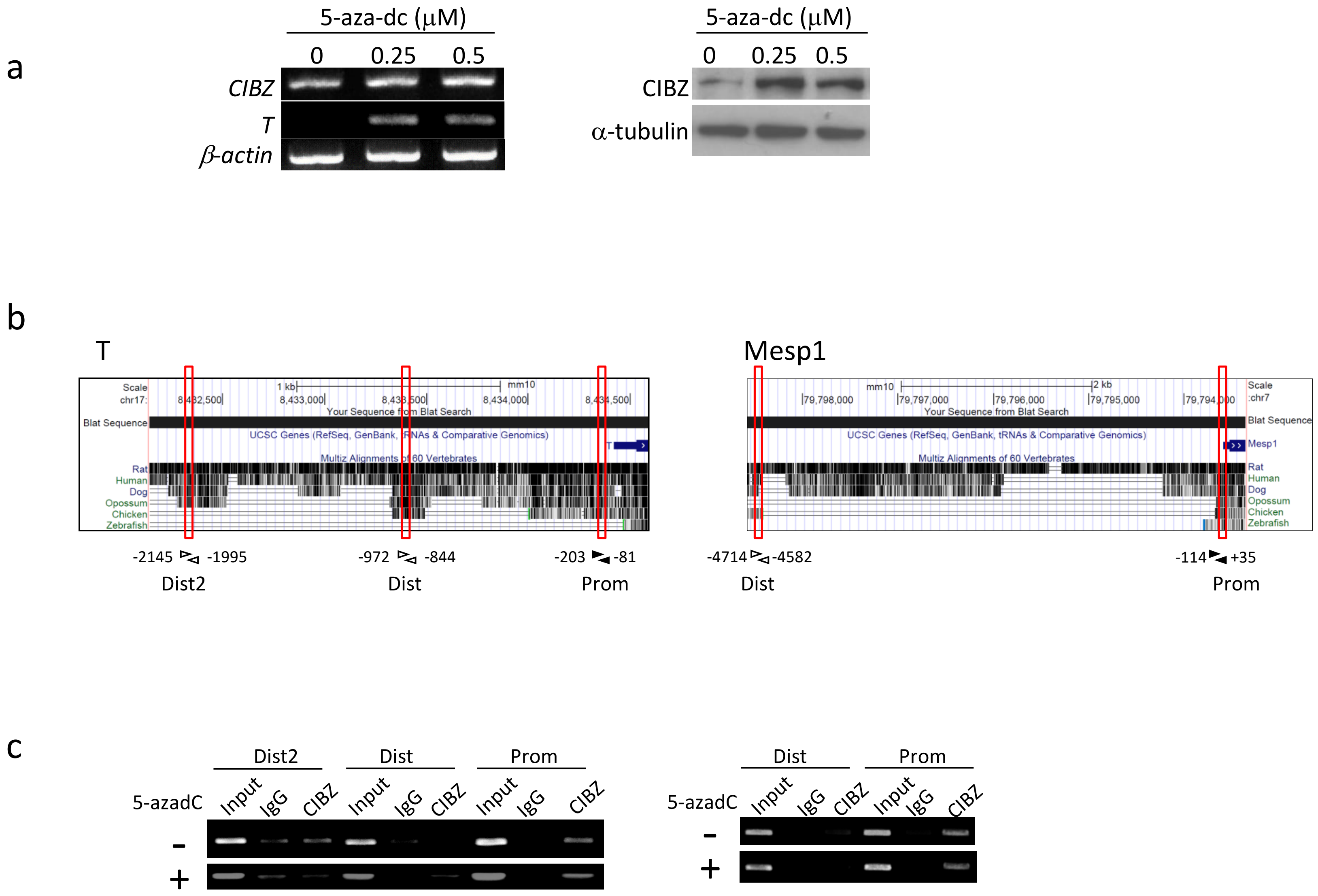

Supplementary Fig. S7

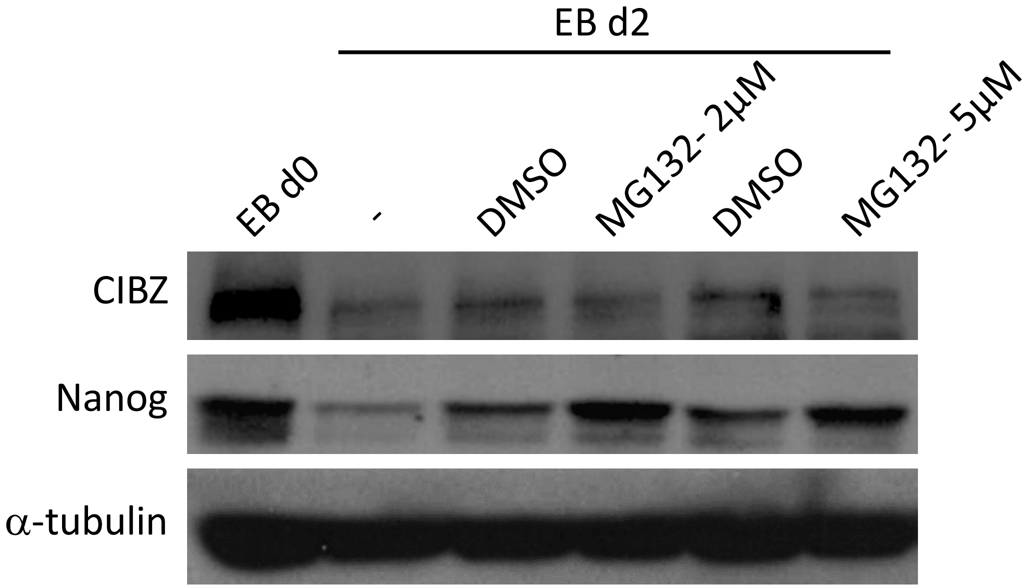

Table S1. Primer sequences for semi-quantitative RT-PCR

Gene	Primer sequence (5′to 3′)	Product size
Oct3/4	F: TCACTCACATCGCCAATCAG<br>R: CCTGTAGCCTCATACTCTTCTC	305 bp
Sox2	F: CTACAGCATGTCCTACTCGC<br>R: CCTCCCAATTCCCTTGTATCTC	351 bp
Nanog	F: TTCAGAAATCCCTTCCCTCG<br>R: AGTAGCAGACCCTTGTAAGC	162 bp
Sox1	F: ATACCGCAATCCCCTCTCAG<br>R: ACAACATCCGACTCCTCTTCC	167 bp
Nestin	F: CTGGAAGTGGCTACATACAGGAC<br>R:AGTCTCAAGGGTATTAGGCAAGG	210 bp
T	F: GAAGTGAAGGTGGCTGTTGG<br>R: ATTTACCTTCAGCACCGGGA	298 bp
Flk1	F: TCGAGCCCTCATGTCTGAAC<br>R: CACTGAGCGATTTCTCCTCAAC	317 bp
Mesp1	F: GTCTGCAGCGGGGTGTCGTG<br>R: CGGCGGCGTCCAGGTTTCTA	189 bp
Gata 6	F: GGGAGAAACTGTGACAATGAC<br>R:ACGAACGCTTGTGAAATGTG	166 bp
Sox17	F: CGAGCCAAAGCGGAGTCTC<br>R: TGCCAAGGTCAACGCCTTC	156 bp
Gata 4	F: GTGAGCCTGTATGTAATGCC<br>R: CTGTGCCCATAGTGAGATGAC	274 bp
MEF2C	F: GAGATGCCAGTTACCATCCC<br>R: CTTGTTCAGGTTACCAGGTGAG	280 bp
Nkx2.5	F: CCAAGTGCTCTCCTGCTTTCC<br>R: GCCATCCGTCTCGGCTTT	149 bp
Tbx5	F: AGGAGCACAGCCAAATTTACCAC<br>R: ATGAGCGGAGAAGTGCTGGTAG	294 bp
Isl1	F: ATGATGGTGGTTTACAGGCTAA<br>R: TCGATGCTACTTCACTGCCAG	174 bp
cTnI	F: TGCCAACTACCGAGCCTATG<br>R: TGGCAACGAGTCCTCAGAAC	174 bp
GAPDH	F: CCATCACCATCTTCCAGGAG<br>R: CCTGCTTCACCACCTTCTTG	577 bp

Table S2. Primer sequences for qPCR

Gene	Primer sequence (5′ to 3′)	Product size
T	F: GCTTCAAGGAGCTAACTAACGAG<br>R: CCAGCAAGAAAGAGTACATGGC	117 bp
Flk1	F: CTGGAGCCTACAAGTGCTCG<br>R: GAGGTTTGAAATCGACCCTCG	179 bp
Mesp1	F: GTCTGCAGCGGGGTGTCGTG<br>R: CGGCGGCGTCCAGGTTTCTA	189 bp
Nkx2.5	F: GGTCTCAATGCCTATGGCTAC<br>R: GCCAAAGTTCACGAAGTTGCT	153 bp
Gata4	F: AACGGAAGCCCAAGAACCTG<br>R: AGTGGCATTGCTGGAGTTACC	107 bp
Tbx5	F: AATGGTCCGTAACTGGCAAAG<br>R: GGATAATGTGTCCAAACGGGTC	159 bp
Isl1	F: ATGATGGTGGTTTACAGGCTAAC<br>R: TCGATGCTACTTCACTGCCAG	174 bp
Mef2C	F: AGGATAATGGATGAGCGTAACAG<br>R: GTTCAATGCCTCCACAATGTC	240 bp
cTnI	F: TGCCAACTACCGAGCCTATG<br>R: TGGCAACGAGTCCTCAGAAC	174 bp
MHC	F: CAGAGGAGAAGGCTGGTGTC<br>R: TTGTCAGCATCTTCTGTGCC	121 bp
GAPDH	F: CAATGTGTCCGTCGTGGATCT<br>R: GTCCTCAGTGTAGCCCAAGATG	124 bp

Table S3. Primers for ChIP assay

	Primer sequence (5′ to 3′)
T<br>(Promoter region)	F: GCTGCTCGGTACTTCAAAGGG<br>R: GCGCGACAAGAGTAAGTCTCTG
T<br>(Distal region)	F: TCCTGCTCTTTGTCACCTTC<br>R: GATTGTTGGAACGCATGCTG
Mesp1<br>(Promoter region)	F: GTGGAGCAGACTGGACTAAG<br>R: TTATCCTGAGCCCTAGGTGTG
Mesp1<br>(Distal region)	F: ACTCTAGCTGCCTGTCTTGG<br>R: CCTTACTTCACATACCAGAGCCTT
Flk1<br>(Promoter region)	F: GACTTTCAGTGCAGCGGCGAAG<br>R: CAAATCTGGACGCAGCTCGGTTTC
Gata4<br>(Promoter region)	F: CGTAGATCTGAGGCTAGCAAGGC<br>R: CTCTTTCCTCCCTACTCTCAGTGGTC
Nkx2.5<br>(Promoter region)	F: CTGGCTGGGATTTTCAGGCTAACGAG<br>R: ACGGGCAGTTCTGCGTCACCTAAT

Table S4. Primers for pGL3-T and pGL-3-Mesp1 constructs

	Primer sequence (5′ to 3′)
T	F: AGACGACGCGTCAAAGTCGCAGGCGCCGGTGTG<br>R: GTCCCAAGCTTCCACCCTCTCCACCTTCCAG
Mesp1	F: AGACGACGCGTCAAGGCTCTGGTATGTGAAGTAAGG<br>R: GTCCCAAGCTTGGCAGCGGAGGCCTGACCATTG

Supplementary video legends

Supplementary video 1. Representative video documenting the spontaneous beating of WT EBs at day 10 of ESC differentiation.

Supplementary video 2. Representative video documenting the spontaneous beating of CIBZ$^{-/-}$ EBs at day 10 of ESC differentiation.
